# Effect of D1- and D2-like Dopamine Receptor Antagonists on the Rewarding and Anxiolytic Effects of Neurotensin in the Ventral Pallidum

**DOI:** 10.3390/biomedicines10092104

**Published:** 2022-08-28

**Authors:** Tamás Ollmann, László Lénárd, László Péczely, Beáta Berta, Erika Kertes, Olga Zagorácz, Edina Hormay, Kristóf László, Ádám Szabó, Rita Gálosi, Zoltán Karádi, Veronika Kállai

**Affiliations:** 1 Institute of Physiology, Medical School, University of Pécs, H-7624 Pécs, Hungary; 2 Centre for Neuroscience, University of Pécs, H-7624 Pécs, Hungary; 3 Molecular Neuroendocrinology and Neurophysiology Research Group, Szentágothai Center, University of Pécs, H-7624 Pécs, Hungary

**Keywords:** neurotensin, reward, anxiety, ventral pallidum, D1- and D2-like dopamine receptors, conditioned place preference, elevated plus maze test

## Abstract

Background: Neurotensin (NT) acts as a neurotransmitter and neuromodulator in the central nervous system. It was shown previously that NT in the ventral pallidum (VP) has rewarding and anxiolytic effects. NT exerts its effect in interaction with dopamine (DA) receptors in numerous brain areas; however, this has not yet been investigated in the VP. The aim of this study was to examine whether the inhibition of D1-like and D2-like DA receptors of the VP can modify the above mentioned effects of NT. Methods: Microinjection cannulas were implanted by means of stereotaxic operations into the VP of male Wistar rats. The rewarding effect of NT was examined by means of a conditioned place preference test. Anxiety was investigated with an elevated plus maze test. To investigate the possible interaction, D1-like DA receptor antagonist SCH23390 or D2-like DA receptor antagonist sulpiride were microinjected prior to NT. All of the drugs were also injected independently to analyze their effects alone. Results: In the present experiments, both the rewarding and anxiolytic effects of NT in the VP were prevented by both D1-like and D2-like DA receptor antagonists. Administered on their own, the antagonists did not influence reward and anxiety. Conclusion: Our present results show that the activity of the D1-like and D2-like DA receptors of the VP is a necessary requirement for both the rewarding and anxiolytic effects of NT.

## 1. Introduction

Drug addiction and anxiety disorders are among the most serious social and health problems in our world today [1]. Due to the above, both addiction and anxiety disorders are at the forefront of recent clinical research. Dopamine (DA) [2,3] and neurotensin (NT) [4,5,6] as well as their interactions [7] play a significant role in the pathomechanism of addiction and anxiety.

DA is a neurotransmitter that belongs to catecholamines [8]. The mesolimbic DAergic system originating from the ventral tegmental area (VTA) is involved in the regulation of stress, anxiety, and reward-related behaviour. The regulation of these processes are interconnected, i.e., aversive or stressful events can negatively regulate the DAergic reward system [8].

The ventral pallidum (VP) is the ventral extension of the globus pallidus [9], and it is innervated by mesolimbic DAergic fibres originating from the VTA [10]. The role of DA receptors of the VP has also been demonstrated in rewarding and reinforcement processes [11,12]. Both D1-like (D1 and D5) and D2-like (D2, D3, as well as D4) subgroups of DA receptors were previously detected in the VP [13]. D1-like DA receptors are localized both pre- and postsynaptically [14,15,16], and D2-like DA-receptors are localized mainly presynaptically on the ventral striatopallidal GABAergic fibres, and in a low amount (as autoreceptors) in the DAergic axon terminals originating from the VTA, as well as on the projection neurons and interneurons of the VP [17].

NT in the central nervous system also plays an important role both in rewarding processes [4,7,18,19,20], and in the regulation of anxiety [5,21,22]. NT is a tridecapeptide (pGlu-Leu-Tyr-Glu-Asn-Lys-Pro-Arg-Arg-Pro-Tyr-Ile-Leu-OH) originally isolated by Robert Carraway and Susan E. Leeman [23]. NT acts as a neurotransmitter and a neuromodulator, inter alia in the mesolimbic DAergic system [24]. The effects of NT are mediated through at least three different receptors (NTS1, NTS2 and NTS3), and it modulates the effect of DA in numerous brain areas, i.e., in the VTA, nucleus accumbens (NAC), amygdala, hippocampus, entorhinal and piriform cortex [25,26,27].

VP is also innervated by NTergic fibres, which mainly travel from the NAC through the ventral striatopallidal GABAergic pathway [28,29]. NTergic axon terminals and NT-immunoreactivity are localized in the ventromedial subregion of the VP (VPvm), however, they cannot be detected in other subregions of the VP [28,29,30]. Earlier experiments of our research group showed that NT microinjected into the VP has rewarding [31] and anxiolytic [32] effects through NTS1-receptors.

Since NT is considered as a neuromodulator rather than a simple neurotransmitter, it can be assumed that it exerts its effect by modifying the function of other neurotransmitters. As mentioned above, both NT and DA receptors can be found in the VP. Additionally, DA receptors can be detected on the axon terminals of NTergic fibres travelling in the ventral striatopallidal pathway. Based on these factors, functional cooperation can be plausible between the two systems. Indeed, interactions between NTergic and DAergic receptors were characterized in the above mentioned brain regions, i.e., VTA, NAC, amygdala, hippocampus, entorhinal and piriform cortex [25,26,27]. Thus, we can hypothesize that such an interaction can also operate in the VP, which may underlie the effects of NT, but this has not been investigated yet. Therefore, in the present experiments, we examined the role of D1-like and D2-like DAergic receptors in the rewarding or anxiolytic effects of NT in the VP.

## 2. Materials and Methods

### 2.1. Animals

Experiments were carried out on 165 male Wistar rats (LATI, Gödöllő) weighing 280–320 g. In the present experiments only male rats were used to avoid the influence of cyclic hormonal changes on the behavioural patterns. Animals were held in a temperature (22 ± 1 °C, air humidity: 55 ± 10%) and light controlled room. Artificial illumination was applied according to the natural daylight period, with a 12:12 h light-dark cycle. The daylight period started at 6:00, and the dark period started at 18.00. The animals were cared for in accordance with national (Hungarian Government Decree, 40/2013. (II. 14.)), international (European Community Council Directive, 86/609/EEC, 1986, 2010) and institutional standards (number of ethical permissions: BA02/2000-73/2017, BA02/2000-74/2017).

### 2.2. Surgery

During stereotaxic operations, general anaesthesia with intraperitoneal injection of ketamine (Calypsol, Richter Gedeon, Hungary, 80 mg/kg body weight) and diazepam (Seduxen, Richter Gedeon, Hungary, 20 mg/kg body weight) was applied. By means of the stereotaxic technique, 22-gauge (0.64 mm) stainless steel guide tubes were implanted 0.5 mm above the VP on both sides. The coordinates were determined according to the stereotaxic rat brain atlas of Paxinos and Watson [33]. The following coordinates referring to the bregma were used: anteroposterior (AP): −0.26 mm; mediolateral (ML): 2.2 mm; and dorsoventral (DV): 7.1 mm from the surface of the dura. The cannulas were fixed with self-polymerizing dental acrylic (Duracryl) to 3 stainless steel screws that were screwed into the skull. The guide tubes were occluded with sterile 27-gauge (0.36 mm) stainless steel obturators which were removed during microinjections. Antibiotic prophylaxis (penicillin G) was applied during the operations. Before starting the behavioural experiments, animals were provided with a minimum of 6 days for postoperative recovery. During the recovery period, handling of the animals was performed daily by the experimenters. Neurological examinations were carried out on all animals to verify the intact sensory and motor functions. Behavioural tests were performed during the daylight period between 08:00 and 18:00. The animals were randomly divided into the groups. The experimenters were not blinded during the behavioural tests since all of the parameters were analyzed by the computer program independently of the experimenters. During the histological analysis, the experimenters were blinded for both the treatment and results of the experiments.

### 2.3. Drugs

The NT (Sigma–Aldrich Co., St. Louis, MO, USA, N 6383) and the D1-like DA receptor antagonist (R)-(+)-SCH23390-hydrochloride (Sigma-Aldrich Co., St. Louis, MO, USA, D054) were dissolved in a 0.15 M sterile saline solution containing 0.01 M Na-acetate and 0.01 M phosphate-buffered saline. The D2-like DA receptor antagonist (S)-(-)-sulpiride (Sigma–Aldrich Co., S7771) was dissolved in 0.1 N HCl, and after the addition of the phosphate buffer (pH 6.9) it was titrated with 0.1 N NaOH. NT was microinjected at a dose of 100 ng, SCH23390 at 1 μg and sulpiride at a dose of 4 μg. The dose of NT was determined based on the effective dose of NT in the VP in the earlier behavioural experiments conducted by our research group [31,32]. The doses of the DA receptor antagonists were determined based on pilot experiments and on effective dose ranges blocking the effect of NT in other brain areas [27]. Control rats received the corresponding vehicle solution in the same volume (0.4 μL). All mentioned doses were applied per side.

### 2.4. Microinjection Procedure

The microinjections were applied on awake rats who were gently held by the experimenters. All drugs or vehicles were bilaterally microinjected through 27-gauge stainless steel microinjection tubes extending 0.5 mm below the tips of the implanted guide cannulae. The delivery cannulae were attached to a 10 μL Hamilton microsyringe (Hamilton Co., Bonaduz, Switzerland) via polyethylene tubes (PE-10). All injections were delivered by a Cole-Parmer 74900-15 digital infusion pump at a volume of 0.4 μL (Cole Parmer, IITC, Life Sci. Instruments, Woodland Hills, Los Angeles, CA, USA) over a 60 s interval. After accomplishing the microinjection, cannulae were left in place for an additional 60 s for diffusion into the surrounding tissue. After all injections it was verified that the cannulae were not occluded.

The effect of D1-like or D2-like DA receptor antagonism was examined both in conditioned place preference (CPP) and elevated plus maze (EPM) paradigms; thus, the experiments used 4 cohorts of animals. According to the drug treatment, each cohort was divided into 4 groups. During the investigation of the blocking effect of antagonists, a combined treatment was necessary whereby the rats first received the microinjection of D1-like or D2-like DA receptor antagonist followed by NT. Nevertheless, the double volume exposure could affect the data compared to single volume injection in other treatment groups. Therefore, to exclude this methodological failure, all rat groups received double microinjections. The placebo injection included vehicle. So, the groups were as follows: 1. The control group received two vehicle injections; 2. the NT group was microinjected first with vehicle and then with 100 ng NT; 3. the antagonist group received first the antagonist (1 μg SCH23390 or 4 µg sulpiride) and then vehicle; and 4. the combined treatment group was first pretreated with antagonist (1 μg SCH23390 or 4 µg sulpiride) and then with 100 ng NT.

The two microinjections were applied 15 min after each other, and then rats were placed into the arena. The experimental cohorts as well as the number of animals involved in the statistical analysis (145 out of 165 animals) are summarized in Table 1.

### 2.5. Behavioural Testing

All of the experiments were carried out in a sound-proof and dimly illuminated room (by a 40 W bulb). The events were recorded by a video camera, and the data were analyzed off-line using the Noldus EthoVision Basic 2.3 video tracking system (EthoVision; Noldus Information Technology, Wageningen, The Netherlands). The position of the animal was determined based on the central point of the animal in each of the experiments. Entry to the quadrants or arms was defined as entry of the central point.

#### 2.5.1. Conditioned Place Preference (CPP) Test

The CPP test can be used to test the rewarding, positive reinforcing or aversive, punishing effects of drugs [34,35]. The CPP apparatus was a plastic grey colored tub, containing a circular 85 cm diameter floor, encircled by a 40 cm high wall. The floor was divided by thin black lines into four quadrants, which were separated from each other by removable plexiglas barriers during conditioning. Various visual cues were placed around the apparatus to assist the animals in spatial orientation within the device and in distinguishing quadrants.

The CPP paradigm was composed of one habituation (1st day), three conditioning (2nd–4th days) trials and one test (5th day) trial. Each lasted for 900 s (15 min). In the habituation trial (1st day), animals had free access to all quadrants of the apparatus. The time that the rats had spent in each of the four quadrants during habituation was measured. Based on this, we determined the treatment quadrant, which was one of the quadrants in which the animal spent neither the longest nor the shortest time [34,36]. In the habituation trial, there was no initial quadrant preference that can be explained by the homogenous environment. During the conditioning trials (2nd–4th days), the quadrants were physically separated from each other by plexiglas barriers. Following bilateral microinjections, animals were placed into the treatment quadrant, and they spent 15 min in this quadrant. On the 5th day (test trial), the separating barriers were removed, i.e., animals again had free access to all parts of the arena. The time spent in each of the four quadrants was measured. In relation to the rewarding effect, increased time spent in the treatment quadrant was observed.

#### 2.5.2. Elevated plus Maze (EPM) Test

An EPM test was performed to examine anxiety [37]. The apparatus consisted of grey painted wooden boards assembled in a cross shape. Two opposite boards were surrounded by a 40 cm high wall which formed the closed arms, while the other two boards had no wall—these were the open arms. Each arm had a floor area of 50 cm × 10 cm, the area of the centre of the maze (central platform) was 10 cm × 10 cm, and the roof was open. The maze was built on a scaffold that was 100 cm high. Immediately after microinjections, animals were placed into the central platform facing one of the closed arms. The test was performed once on each animal, and it lasted for 5 min. The time spent at the different parts of the apparatus (open and closed arms and the end of the open arms) was measured. In case of the anxiolytic effect, increased time spent at the open arms and at the end of the open arms was detected.

### 2.6. Histology

On completion of the behavioural examinations, rats were overdosed with intraperitoneal urethane injection (20% urethane solution in a dose of 1.4 g/kg body mass) and then perfused transcardially with a physiological saline solution followed by a 10% formaldehyde solution. A week after tissue fixation, brains were cut with a freezing microtome into 40 μm coronal sections and then the sections were stained with Cresyl-violet. Using a light microscope, cannula tracks were observed and the location of the cannula ends was assessed based on the stereotaxic atlas of the rat brain. [38]. Only data from animals were included in the data analysis in which the cannulae were in the appropriate location. those

### 2.7. Statistical Analysis

Normal distribution of the data was verified by performing a Shapiro–Wilk-test, and then the data were evaluated with a one-way or mixed analysis of variance (ANOVA) by means of the “SPSS 20.0 for Windows” program. The homogeneity of the sample was examined by performing an F-test. Bonferroni’s post hoc test was employed for the comparison of the groups. Statistical significance was established at *p* < 0.05.

## 3. Results

### 3.1. Histology

**Figure 1:**
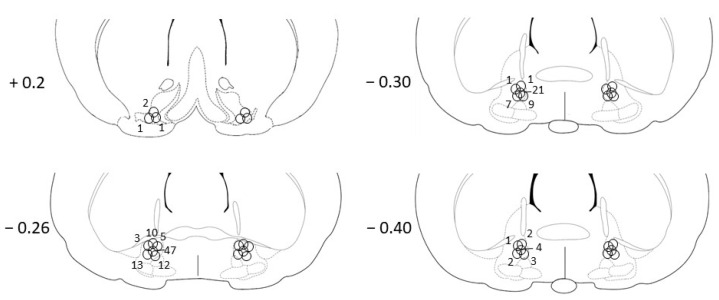
Illustration of correct bilateral injection placements of the injector tips (*n* = 145). The numbers on the left side of the coronal brain sections refer to anterior–posterior distance from the bregma in mm. The borders of VP are presented based on the stereotaxic rat brain atlas of Paxinos and Watson [38]. The circular symbols indicate the centre of the bilateral injection sites. Numbers next to the symbols indicate the number of animals with similar cannula positions.

The cannula tracks and the tip positions were determined on the basis of the stereotaxic atlas of Paxinos and Watson [38]. Four of the 165 animals died before the end of the experiments. The histological examination showed that the cannulae were precisely and symmetrically tipped to the target area in 145 animals (Figure 1). For 16 animals (~10%), mistargeted cannula placement was verified, and data of these animals were excluded from subsequent analysis. Since in the mistargeted cases cannulae were located at different brain areas, and the animals were also distributed between the four experiments, no far-reaching conclusions could be drawn from these data. Only data from animals (*n* = 145) where the cannula was placed in the VP were considered in the analysis.

### 3.2. Conditioned Place Preference Test

**Figure 2:**
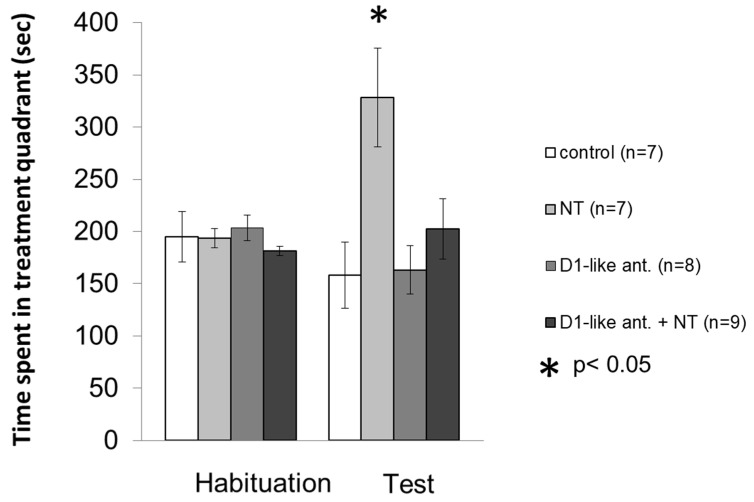
Effects of D1-like DA receptor antagonist SCH23390 pretreatment on the NT induced conditioned place preference in the VP. Columns represent mean time spent in the treatment quadrant (±S.E.M.) during Habituation and Test trials, respectively. Control group: rats received two microinjections of vehicle (n = 7). NT group: animals microinjected with vehicle + 100 ng NT (n = 7). D1-like ant. group: animals received 1 µg SCH23390 + vehic1e (n = 8). D1-like ant. + NT group: effect of microinjections of 100 ng NT after 1 µg SCH23390 pretreatment (n = 9). For further explanation, refer to the text. *: *p* < 0.05.

The aim of the first experiment was to investigate whether the interaction with D1-like DA receptors plays a role in the place preference inducing effect of 100 ng NT (Figure 2).

The mixed ANOVA did not show a significant difference between the trials (F [1; 31] = 1.428, *p* > 0.05), but a significant effect was detected between the treatments (F [3; 31] = 4.978, *p* < 0.01), and in the case of the interaction between trials and treatments (F [3; 31] = 5.749, *p* < 0.01). According to Bonferroni’s post hoc test, the time spent in the treatment quadrant in the test trial increased in the group treated with 100 ng NT (n = 7) compared to the control group (*p* < 0.05, n = 7), the D1-like DA receptor antagonist treated group (*p* < 0.05, n = 8) and animals receiving 100 ng NT after pretreatment with D1-like antagonist (*p* < 0.05, n = 9). The D1-like antagonist on its own did not influence the time spent in the treatment quadrant, i.e., the result did not differ from the control group.

**Figure 3:**
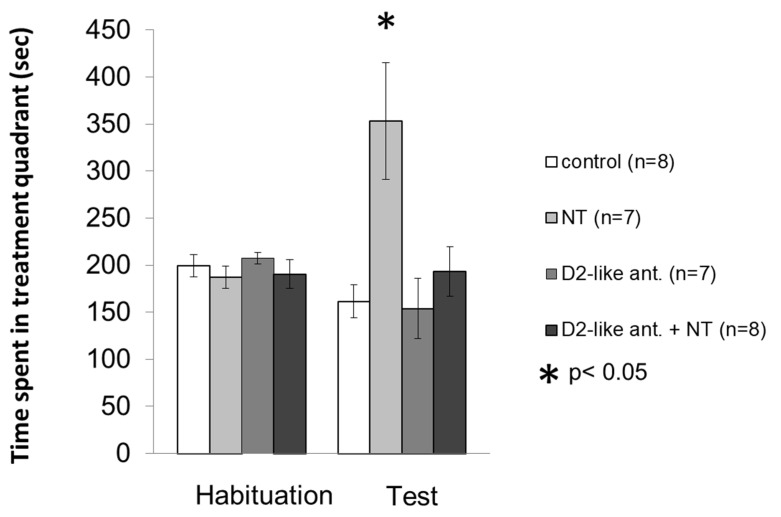
Effects of D2-like DA receptor antagonist sulpiride pretreatment on the NT induced conditioned place preference in the VP. Columns represent mean time spent in the treatment quadrant (±S.E.M.) during Habituation and Test trials, respectively. Control group: rats received two microinjections of vehicle (n = 8). NT group: animals microinjected with vehicle + 100 ng NT (n = 7). D2-like ant. group: animals received 4 µg D2-like DA receptor antagonist sulpiride + vehic1e (n = 7). D2-like ant. + NT group: effect of microinjections of 100 ng NT after 4 µg sulpiride pretreatment (n = 8). For further explanation, refer to the text. *: *p* < 0.05.

The results of NT-sulpiride (antagonist of D2-like DA receptors) interactions in the CPP experiment are shown in Figure 3.

Mixed ANOVA revealed no significant difference between the trials (F [1; 30] = 1.134, *p* > 0.05), but the test showed a significant difference between the treatments (F [3; 30] = 5.791, *p* < 0.01), and in the interaction between trials and treatments (F [3; 30] = 7.249, *p* ≈ 0.001). Based on Bonferroni’s post hoc test, the time spent in the treatment quadrant in the test trial increased in the 100 ng NT treated group (n = 7) relative to all groups: the control group (*p* < 0.01, n = 8), the D2-like DA receptor antagonist treated group (*p* < 0.01, n = 7), and animals receiving 100 ng NT after pretreatment with D2-like antagonist (*p* < 0.05, n = 8). Based on these results, we found that the sulpiride pretreatment blocked the effect of NT. The sulpiride by itself did not influence the time spent in the treatment quadrant, i.e., the result did not differ from the control group.

### 3.3. Elevated plus Maze Test

**Figure 4:**
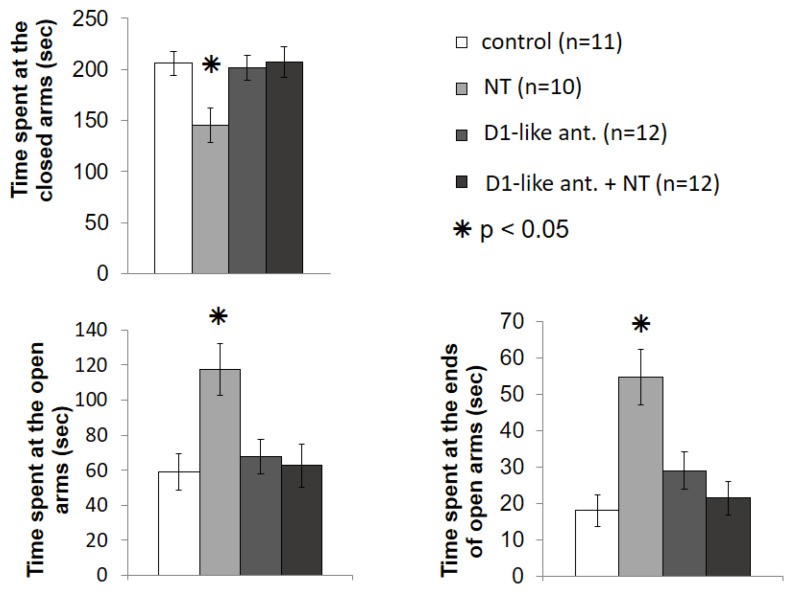
Effects of D1-like DA receptor antagonist SCH23390 pretreatment on the anxiolytic effect of NT in the VP in EPM test. Columns represent mean time spent at the closed arms, time spent at the open arms, and time spent at the ends of the open arms, respectively (±S.E.M.). Control group: rats received two microinjections of vehicle (n = 11). NT group: animals microinjected with vehicle + 100 ng NT (n = 10). D1-like ant. group: animals received 1 µg SCH23390 + vehicle (n = 12). D1-like ant. + NT group: effect of microinjections of 100 ng NT after 1 µg SCH23390 pretreatment (n = 12). For further explanation, refer to the text. *: *p* < 0.05.

In the first EPM experiment, we examined whether D1-like DA receptors play a role in the anxiolytic effect of NT in the VP (Figure 4).

Based on one-way ANOVA, there was a significant difference in the time spent at closed arms (F [3; 41] = 4.120; *p* < 0.05), time spent at open arms (F [3; 41] = 4.976; *p* < 0.01), as well as time spent at the ends of open arms (F [3; 41] = 8.555; *p* < 0.001). Bonferroni’s post hoc test revealed that the SCH23390 in itself (n = 12) did not influence the time spent in each arm, as there was no difference compared to the control group (n = 11). In the group treated with 100 ng NT (n = 10), the time spent at the open arms was significantly higher (*p* < 0.01 relative to the control and *p* < 0.05 relative to the other groups). Similarly, in the NT treated group, time spent at the ends of open arms was significantly higher (*p* < 0.001 relative to the control, *p* ≈ 0.001 relative to D1-like ant. + NT, and *p* < 0.05 relative to D1-like ant.). The time spent at closed arms was significantly lower in the NT treated group than in the other groups (*p* < 0.05 relative to the control and D1-like ant. + NT group, and *p* ≈ 0.05 relative to the D1-like ant. group). The results of the group treated with NT following D1-like DA receptor antagonist pretreatment (n = 12) were significantly different from those of the 100 ng NT-treated animals, but not from the control group in each arm. Based on this, it can be stated that D1-like DA receptor antagonist SCH23390 prevented the anxiolytic effect of NT.

**Figure 5:**
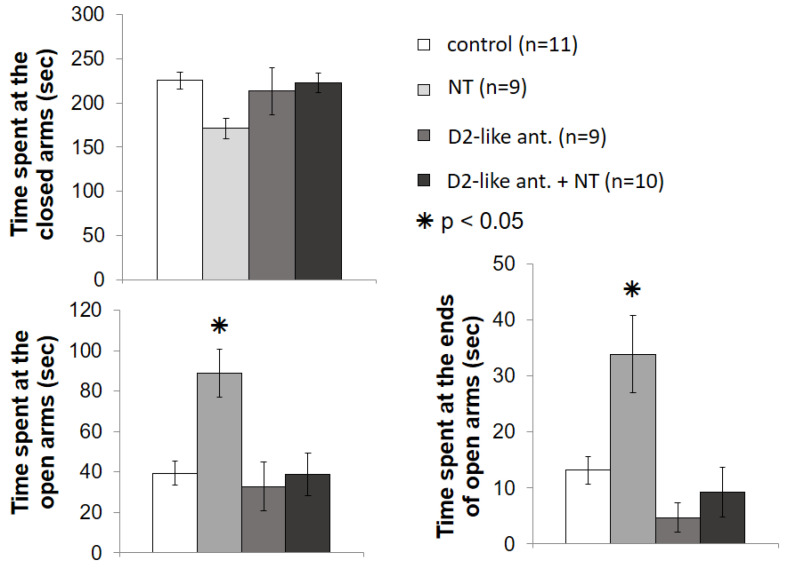
Effects of D2-like DA receptor antagonist sulpiride pretreatment on the anxiolytic effect of NT in the VP in EPM test. Columns represent mean time spent at the closed arms, time spent at the open arms, and time spent at the ends of the open arms, respectively (±S.E.M.). Control group: rats received two microinjections of vehicle (n = 11). NT group: animals microinjected with vehicle + 100 ng NT (n = 9). D2-like ant. group: animals received 4 µg D2-like DA receptor antagonist sulpiride + vehicle (n = 9). D2-like ant. + NT group: effect of microinjections of 100 ng NT after 4 µg sulpiride pretreatment (n = 10). For further explanation, refer to the text. *: *p* < 0.05.

The results of the EPM experiment regarding interactions of D2-like DA receptors and NT are shown in Figure 5.

The one-way ANOVA showed no significant difference among the groups in terms of time spent at the closed arms (F [3; 38] = 2.599; *p* > 0.05). However, there was a significant difference in time spent at open arms (F [3; 38] = 6.306; *p* < 0.01), and at the ends of open arm F [3; 38] = 8.177; *p* < 0.001). The Bonferroni’s post hoc test revealed that sulpiride in the applied dose did not influence the time spent at the different arms. In the sulpiride treated group (n = 9), although the average time spent at the ends of open arms was somewhat shorter, none of the measured times were significantly different from the control group (n = 11). The animals treated with 100 ng NT (n = 9) spent significantly more time at the open arms (*p* < 0.01 relative to all other groups), and at the ends of the open arms (*p* < 0.001 relative to the D2-like ant. group, *p* < 0.01 relative to the D2-like ant. + NT group and *p* ≈ 0.01 relative to the control group). The results of the NT injected group pretreated with sulpiride (n = 10) did not significantly differ from the results of control group, but the mice included in this group spent a significantly shorter time at the open arms, and at the ends of the open arms, than the 100 ng NT treated animals. Consequently, the D2-like DA receptor antagonist sulpiride pretreatment prevented the anxiolytic effect of NT.

## 4. Discussion

The main questions of the present experiments were whether the rewarding and anxiolytic effect of NT microinjected into VP could be modified or inhibited by DA receptor antagonists microinjected into VP.

The VP plays a crucial role in CPP, since in the case of lesions of VP, cocaine, amphetamines or sucrose-induced place preference cannot be evoked [12,39,40]. Our research team previously demonstrated that the rewarding effect of NT microinjected into VP is mediated through NTS1 receptors [31]. The role of DA in the regulation of synaptic plasticity [41] and in reward processes [8] is well-known; therefore, we supposed that the NT may exert its effect directly or indirectly via the DAergic system. Our present results support this hypothesis since the rewarding effect of NT can be prevented by D1-like or D2-like DA receptor antagonists, respectively. Some results in the literature suggest that DA in the VP has a rewarding effect [42,43]. It has been shown that the indirect DA agonist cocaine and amphetamine in the VP can also cause place preference [44]. The intra-VP psychostimulant infusions increase extracellular DA concentration in the VP [44,45] and the 6-hydroxidopamine (6-OHDA) lesion of the VP can block the cocaine induced place preference [12]. Nevertheless, the inhibition of opioid receptors of the VP can abolish or at least attenuate the cocaine evoked place preference [46]. In our present experiments, neither the D1- nor the D2-like DA receptor antagonist induced place preference or aversion by themselves, suggesting that the inhibition of the VP DA receptors does not directly influence reinforcement. Indeed, we already demonstrated that the intra-VP sulpiride impairs learning processes in the Morris Water Maze paradigm [47], suggesting that the D2-like DA receptors of the VP are necessary for the consolidation of memory. Therefore, it is also possible that DA receptor antagonism interferes not with rewarding itself, but rather with the memory consolidation of rewarding processes.

Our second question was whether the anxiolytic effect of NT microinjected into VP could be blocked by the ventral pallidal microinjection of DA receptor antagonists. Our present results show that both D1-like and D2-like DA receptor antagonists can prevent the anxiolytic effect of NT, although the antagonists in themselves do not significantly affect anxiety. Our research group previously demonstrated the anxiolytic effect of 100 ng dose of NT in VP via NTS1 receptors [32]. The effect of NT on anxiety was demonstrated in other brain areas as well, as it has an anxiolytic effect in the NAC following lesion of the dorsal raphe nucleus [48]; however, after the systemic administration of reserpine, it increases anxiety [49]. Based on this, the anxiolytic effect of NT in the NAC appears to be state-dependent and aims at restoring the balance of monoamines [49].

According to our recent hypothesis, the intra-VP microinjection of NT can induce place preference and exert its anxiolytic effects presumably via the VP-VTA axis [7]. It was shown that the activity of the DAergic neurons in the VTA is associated with the rewarding function, and on the other hand, the silencing of these neurons can be observed during aversive processes [50]. The VP is one of the main regulators of the population activity of the DAergic neurons of the VTA [51,52], so it is plausible to suppose that the NT can directly or indirectly inhibit GABAergic output neurons of the VP, disinhibiting the VTA and inducing place preference in this way.

Within VP, NT is present almost exclusively in the ventromedial subregion (VPvm) [28], so this subregion is most likely responsible for the rewarding and anxiolytic effect of NT microinjected into VP. Based on our results, both the rewarding and anxiolytic effects of NT require the activity both of the NTS1-receptors [31,32] and the D1-like and D2-like DA receptors of the VP. In human fMRI studies, the VPvm was activated by rotten and unappealing foods, whereas the dorsolateral subregion of the VP responded to the presentation of tasty foods [53]. Based on this, the activation of VPvm should be rather aversive, but its inhibition should be rewarding.

Data in the literature demonstrate that local inactivation of VPvm inhibits the GABAergic efferent projections to the VTA, thereby releasing the VTA from inhibition and increasing the firing rate of DAergic neurons of the VTA. Meanwhile, the extracellular DA level increases in the NAC as well [51,54], which can be associated with reward and positive reinforcement [55,56,57]. The same mechanism is also likely to be responsible for the rewarding and anxiolytic effects of NT microinjected into the VP. This is also supported by the fact that the microinjection of NT into the VP locally increases the extracellular GABA levels [58], presumably by activating fibres originating from the NAC or interneurons of the VP. The GABA, released locally in the VP, inhibits the GABAergic output neurons of the VP innervating the VTA, among others [59]. Therefore, the VTA can be released from inhibition, eliciting both rewarding and anxiolytic effects.

The basolateral amygdala (BLA) can also regulate anxiety through the VP-VTA axis [60]. This is supported by the fact that the decreasing population activity of DAergic neurons of the VTA, caused by chronic, moderate stress, can be attenuated by the local inactivation of the BLA or the VP [61]. NT is also likely to exert its rewarding and anxiolytic effect through modulation of the BLA-VP-VTA axis.

Numerous literary data support the idea that NT exerts its rewarding and anxiolytic effects through modulation of the mesolimbic DAergic system in several different brain regions [24,25,62,63]. NT is colocalized with DA inter alia in the VTA, NAC, amygdala, and prefrontal cortex [64,65,66]. NTS1 and DA receptors were also identified in VP [28,29,67,68]; however, the NT receptors of the VP are located postsynaptically [28,67,69] rather than on DAergic axon terminals, so NT cannot directly modulate the function of DAergic inputs (although a functional interaction between postsynaptic DA and NT receptors can be supposed). D2-like DA receptors in the VP are located primarily presynaptically on GABAergic fibres arising from NAC [17]. D1-like DA receptors can be found both on pre- and postsynaptic sites [14,15,16]. It is most likely that the postsynaptic D1-like DA receptors of VPvm activate the GABAergic interneurons of the VP, and consequently they are able to increase the activity of DAergic neurons in the VTA through the inhibition of projection neurons between VPvm and VTA. Furthermore, the D2-like DA receptors may presynaptically inhibit the release of GABA (primarily arising from axons of the ventral striatopallidal pathway from NAC), contributing to the activation of GABAergic interneurons of the VP.

The present experiments are the first to demonstrate that D1-like and D2-like DA receptor activity in VP are also required for the rewarding and anxiolytic effects of NT. In addition to DA-NT interactions, NT has been shown to modulate the function of GABA in the VP [58]. Furthermore, GABA and DA receptors also interact with each other [70]. Based on this, it can be concluded that although the mechanism of action of NT is likely to involve complex interactions, the endogenous DA activity is a necessary requirement for both rewarding and anxiolytic effects.

Our results provide additional information on the role of DA–NT interactions in rewarding and reinforcement processes, as well as in the regulation of anxiety, that in the future can contribute to the identification of new therapeutical targets in drug addiction and anxiety disorders.

## 5. Conclusions

The activity of D1-like and D2-like DA receptors of the VP is a necessary requirement for both the rewarding and anxiolytic effects of NT, because both effects could be prevented by D1-like DA receptor antagonist SCH23390 or D2-like DA receptor antagonist sulpiride.

## Figures and Tables

**Table 1:** Experimental groups and number of animals (n) in the conditioned place preference (CPP) and elevated plus maze (EPM) test.

	Treatment and Dose		Number of Animals in CPP Test (n)	Number of Animals in EPM Test (n)
	1st Injection	2nd Injection		
**D1-like** **antagonist** **cohorts**	vehicle	vehicle	7	11
	vehicle	100 ng NT	7	10
	1 μg SCH23390	vehicle	8	12
	1 μg SCH23390	100 ng NT	9	12
**D2-like** **antagonist** **cohorts**	vehicle	vehicle	8	11
	vehicle	100 ng NT	7	9
	4 μg sulpiride	vehicle	7	9
	4 μg sulpiride	100 ng NT	8	10

## Data Availability

Not applicable.

## Abbreviations

CPP: conditioned place preference, DA: dopamine, EPM: elevated plus maze, GABA: gamma-aminobutyric acid, NAC: nucleus accumbens, NT: neurotensin, VP: ventral pallidum, VTA: ventral tegmental area.
